# Thrombus aspiration in patients with ST-elevation myocardial infarction: results of a national registry of interventional cardiology

**DOI:** 10.1186/s12872-018-0794-4

**Published:** 2018-04-24

**Authors:** Hélder Pereira, Daniel Caldeira, Rui Campante Teles, Marco Costa, Pedro Canas da Silva, Vasco da Gama Ribeiro, Vítor Brandão, Dinis Martins, Fernando Matias, Francisco Pereira-Machado, José Baptista, Pedro Farto e Abreu, Ricardo Santos, António Drummond, Henrique Cyrne de Carvalho, João Calisto, João Carlos Silva, João Luís Pipa, Jorge Marques, Paulino Sousa, Renato Fernandes, Rui Cruz Ferreira, Sousa Ramos, Eduardo Infante Oliveira, Manuel de Sousa Almeida, on behalf of the investigators of Portuguese Registry on Interventional Cardiology (Registo Nacional de Cardiologia de Intervenção)

**Affiliations:** 1 Serviço de Cardiologia, Hospital Garcia de Orta EPE, Avenida Prof. Torrado da Silva, 2801-951 Almada, Portugal; 2 Centro Cardiovascular da Universidade de Lisboa (CCUL), CAML, Faculdade de Medicina, Universidade de Lisboa, Avenida Professor Egas Moniz, Lisboa, 1649-028 Portugal; 3 Unidade de Farmacologia Clínica, Instituto de Medicina Molecular; Laboratório de Farmacologia Clínica e Terapêutica, Faculdade de Medicina, Universidade de Lisboa, Avenida Professor Egas Moniz, Lisboa, 1649-028 Portugal; 4 Hospital de Santa Cruz, Centro Hospitalar de Lisboa Ocidental, EPE, Lisboa, Portugal; 5 Registo Nacional de Cardiologia de Intervenção, APIC-CNCDC, Lisboa, Portugal; 6 Centro Hospitalar e Universitário de Coimbra – CHC, Coimbra, Portugal; 7 Hospital de Santa Maria, Centro Hospitalar de Lisboa Norte EPE, Lisboa, Portugal; 8 Centro Hospitalar de Vila Nova de Gaia/Espinho - Hospital Eduardo Santos Silva, Porto, Portugal; 9 Hospital de Faro EPE, Faro, Portugal; 10 Hospital do Divino Espírito Santo de Ponta Delgada EPE, Açores, Portugal; 11 Hospital da Cruz Vermelha Portuguesa, Lisboa, Portugal; 12 Hospital da Luz, Lisboa, Portugal; 13 Unidade de Intervenção Cardiovascular – Alvor, Portimão, Portugal; 14 Hospital Professor Doutor Fernando da Fonseca EPE, Amadora, Portugal; 15 Hospital de São Bernardo, Centro Hospitalar de Setúbal EPE, Setúbal, Portugal; 16 Hospital do Funchal, Madeira, Portugal; 17 Hospital de Santo António, Centro Hospitalar do Porto, Porto, Portugal; 18 Centro Hospitalar e Universitário de Coimbra – HUC, Coimbra, Portugal; 19 Centro Hospitalar de São João EPE, Porto, Portugal; 20 Hospital de São Teotónio, Viseu, Portugal; 21 Hospital de São Marcos, Braga, Portugal; 22 Hospital de Vila Real, Centro Hospitalar de Trás-os-Montes e Alto Douro EPE, Vila Real, Portugal; 23 Hospital do Espírito Santo, Évora, Portugal; 24 Hospital de Santa Marta, Centro Hospitalar Lisboa Central EPE, Lisboa, Portugal; 25 Hospital CUF Infante Santo, Lisboa, Portugal; 26 Hospital de Santa Cruz. CHLO; Departamento de Fisiopatologia Nova Medical School, Lisboa, Portugal

**Keywords:** Thrombectomy, Thrombus aspiration, Mortality, Portugal, Primary PCI, Angioplasty

## Abstract

**Background:**

We aimed to evaluate the impact of thrombus aspiration (TA) during primary percutaneous coronary intervention (P-PCI) in ‘real-world’ settings.

**Methods:**

We performed a retrospective study, using data from the National Registry of Interventional Cardiology (RNCI 2006–2012, Portugal) with ST-elevation myocardial infarction (STEMI) patients treated with P-PCI. The primary outcome, in-hospital mortality, was analysed through adjusted odds ratio (aOR) and 95% confidence intervals (95%CI).

**Results:**

We assessed data for 9458 STEMI patients that undergone P-PCI (35% treated with TA). The risk of in-hospital mortality with TA (aOR 0.93, 95%CI:0.54–1.60) was not significantly decreased. After matching patients through the propensity score, TA reduced significantly the risk of in-hospital mortality (OR 0.58, 95%CI:0.35–0.98; 3500 patients).

**Conclusions:**

The whole cohort data does not support the routine use of TA in P-PCI, but the results of the propensity-score matched cohort suggests that the use of selective TA may improve the short-term risks of STEMI.

## Background

The impact of thrombus aspiration (TA) during primary percutaneous coronary intervention (P-PCI) has been widely discussed in the recent years. The removal of the thrombus before stent deployment has shown to improve myocardial blush grade [1], but evidence has been heterogeneous regarding pragmatic outcomes, namely mortality. Survival benefits were previously supported by the TAPAS trial [1], as well as subsequent meta-analyses [2], including of patient level meta-analysis [3]. Both European and American guidelines for the management of patients with ST-Elevation Myocardial Infarction (STEMI) recommended that routine TA should be considered, based on evidence of moderate robustness (Class IIa, level of evidence B) [4, 5]. However, the results of larger trials did not show significant improvements in the mortality. Moreover, the existing evidence does not exclude the possibility of TA benefit in high-risk patients or in selected cases.

We intended to assess the impact of TA outside of the randomized controlled trial setting, to evaluate whether the findings of randomized controlled trials (RCTs) are similar to those occurring in the ‘real-world’.

## Methods

Data was retrieved from the Registo Nacional de Cardiologia de Intervenção (RNCI, Portuguese Registry on Interventional Cardiology) between January 2006 and December 2012 [6].

This is a continuous, prospective and observational registry, which includes all consecutive patients undergoing coronary angiography in multiple centers with interventional cardiology. Until December 2012 there were overall 58,434 procedures registered.

All included patients gave informed consent for the intervention and data collection for CNCDC (Centro Nacional de Colecção de Dados em Cardiologia from the Portuguese Society of Cardiology; http://www.spc.pt/CNCDC/) and the registry procedures are in accordance with the rules of CNPD (Comissão Nacional de Proteccção de Dados – National Committee of Data Protection; https://www.cnpd.pt/). The registry was approved by the Portuguese Society of Cardiology ethics committee and local ethics committees.

Inclusion criteria was P-PCI within less than 12 h of symptom in the context of persistent (> 30 min) ST-segment elevation or new left bundle branch block [7–9]. Exclusion criteria were facilitated and rescue PCI as well stable coronary disease and non-ST elevation acute coronary syndromes.

Patients were stratified in two groups per the presence or absence of TA in the index procedure. TA was performed at the discretion of the operator. Demographic, clinical, patient management-related characteristics, as well as clinical outcomes were assessed.

Our primary outcome was all-cause mortality during the index hospitalization.

The risk was determined by odds ratio (OR) and 95% confidence intervals (CIs) were calculated for the primary outcome. Differences between patients treated with both TA and P-PCI, and those with PCI-PCI alone were adjusted through multivariable regression analysis.

All demographic information, comorbidities and TIMI flow pre-PCI data were included in a multivariable logistic regression to estimate a propensity score for the likelihood of being treated with thrombus aspiration. Matching was performed in a 1:1 fashion using with a 0.05 calliper width of the propensity score [10].

The analyses were further adjusted to residual confounding through multivariable logistics regression, to derive adjusted OR and 95%CIs. Statistical analyses were performed with SPSS version 19.0 (SPSS Inc., Chicago, IL, USA).

## Results

**Fig. 1:**
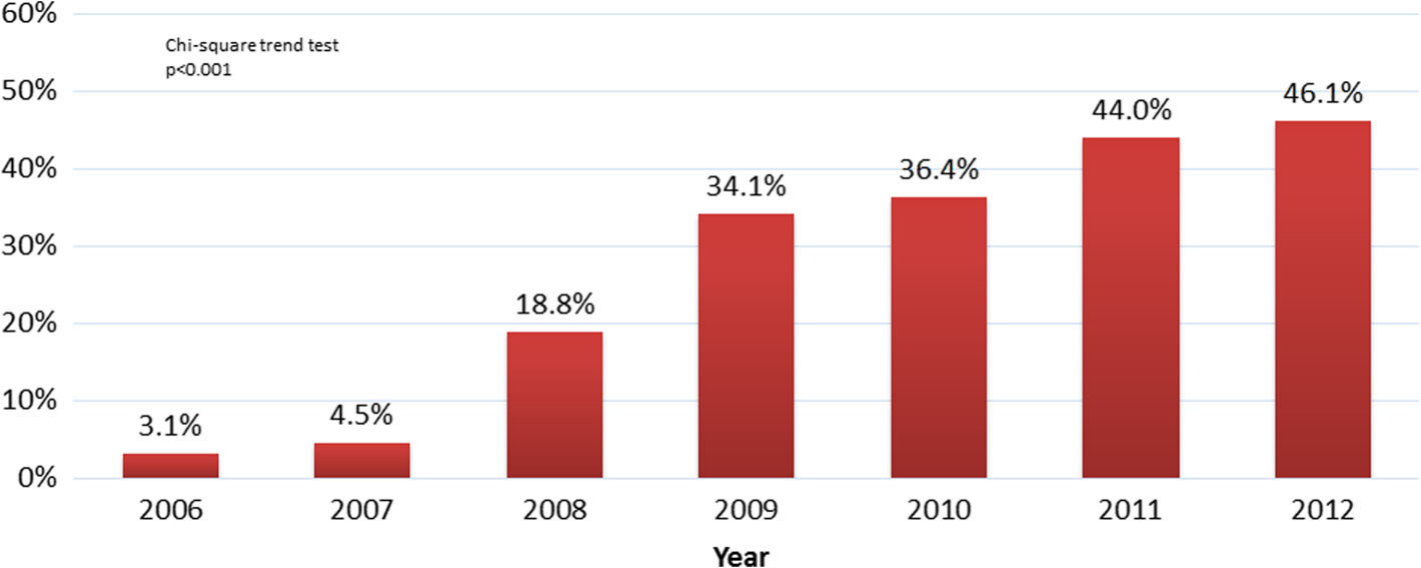
The trend of use of thrombus aspiration in Portugal from 2006 to 2012

A total of 9458 procedures fulfilled the inclusion criteria and 35% of them had adjunctive thrombus aspiration. Time trends show that this procedure had been increasing overtime (Fig. 1).

In the whole cohort, patients treated with TA were overall younger and had lower proportion of cardiovascular risk factors, with exception of smoking, as well as lower proportion of major comorbidities (Table 1). Patients in the group of TA had more frequently a left anterior descending artery occlusion. In the patients with TA the radial access was more common than other accesses, as well as stenting and use of GpIIbIIIa inhibitors (Table 1).

**Table 1:** Clinical characteristics of included patients according to the use of thrombus aspiration during P-PCI

	Before Matching			After Matching		
	Thrombus aspiration and P-PCI (*n* = 3311)	P-PCI only (*n* = 6247)	*p*-value	Thrombus aspiration and P-PCI (*n* = 1750)	P-PCI only (*n* = 1750)	*p*-value
Age	60 ± 13	63 ± 13	< 0.001	61 ± 13	60 ± 13	0.10
Male	77.4%	74.6%	0.002	77.7%	78.6%	0.59
Risk factors						
Dyslipidemia	44.7%	44.6%	0.95	45.0%	46.2%	0.50
DM	18.7%	24.0%	< 0.001	19.0%	18.9%	0.93
Smoking	43.1%	36.4%	< 0.001	41.8%	45.0%	0.38
Hypertension	53.3%	59.7%	< 0.001	54.1%	54.5%	0.87
Previous history						
MI	11.3%	14.2%	< 0.001	12.1%	12.3%	0.84
PCI	12.3%	14.0%	0.035	12.9%	13.8%	0.46
CABG	1.3%	1.7%	0.188	1.3%	1.1%	0.64
Stroke	3.9%	5.2%	0.009	4.0%	4.1%	1.00
PAD	1.9%	3.1%	0.002	1.9%	1.9%	1.00
HF	0.9%	1.9%	< 0.001	0.8%	0.6%	0.69
CKD	3.2%	3.4%	0.56	3.7%	4.6%	0.21
Admission						
KK IV class	6.3%	6.6%	0.57	7.3%	6.4%	0.43
Infarction-related artery						
LM	1.0%	1.3%	0.20	3.6%	3.5%	0.86
LAD	42.4%	35.7%	< 0.001	69.1%	69.4%	0.86
Circumflex	13.4%	17.5%	< 0.001	39.5%	46.8%	< 0.001
RCA	42.8%	45.1%	0.03	58.8%	57.3%	0.36
Radial access	40.2%	22.9%	< 0.001	40.3%	20.3%	< 0.001
Stenting / DES	79.4% / 47.5%	75.9% / 50.9%	< 0.001	91.6% / 45.4%	90.9% / 46.9%	0.50 / 0.40
GpIIb/IIIa	36.9%	18.9%	< 0.001	51.9%	31.0%	< 0.001

*CABG*Coronary artery bypass graft, *CKD* Chronic kidney disease, *DES* Drug-eluting stent, *DM* Diabetes Mellitus, *HF* heart failure, *KK* Killip-Kimball, *LAD* Left Anterior Descending Artery, *LM* Left Main Artery, *MI* Myocardial infarction, *PAD* peripheral artery disease, *PCI* angioplasty/percutaneous coronary intervention, *P-PCI* Primary angioplasty. *RCA* Right Coronary Artery, *TA* Thrombus aspiration

**Fig. 2:**
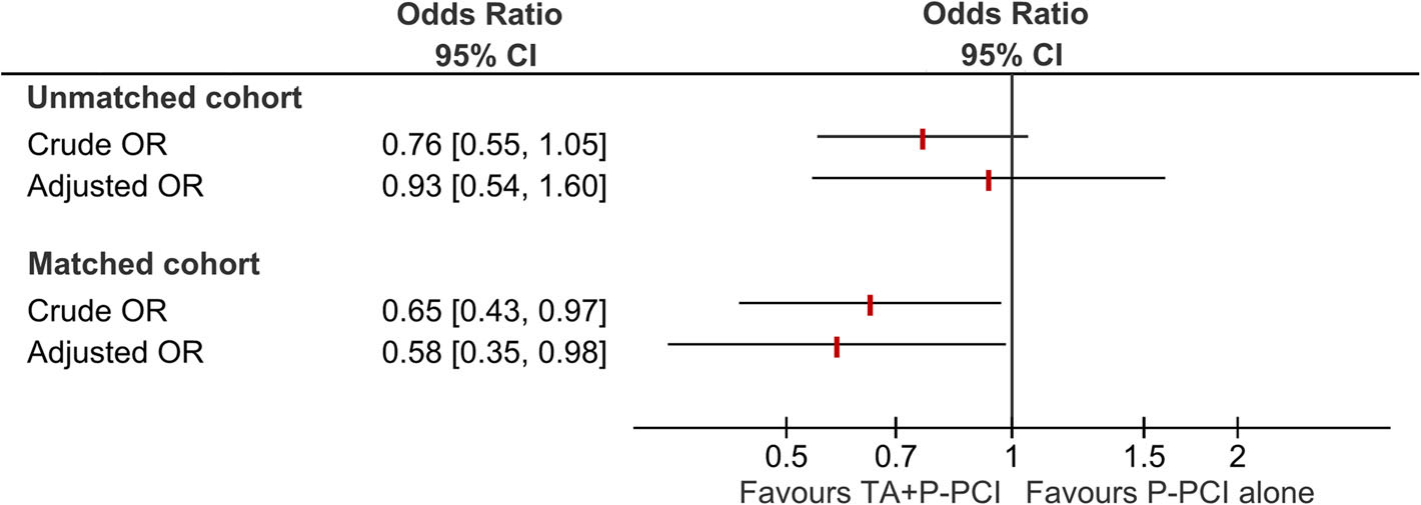
Risk of in-hospital mortality with thrombus aspiration in the whole cohort and in the propensity score matched cohort

The overall in-hospital mortality was non-significantly lower (2.2% vs 2.8%) in the TA group (Fig. 2). Differences between important baseline characteristics were adjusted to confounders and the estimate of TA impact on mortality remained non-significant (OR 0.93, 95%CI 0.54–1.60, *p* = 0.79) (Fig. 2).

The stroke rate was low and thrombus aspiration was unlikely to affect its risk in our cohort (TA + P-PCI vs. P-PCI: 0.1% vs. 0.2%, *p* = 0.77).

We conducted a sub-analysis among the 7.692 patients that full fielded the inclusion criteria and had full information about the TIMI flow of coronary arteries. Patients with TA had more frequently post-PCI TIMI flow< 3 compared to those without TA (14.0% vs.10.6%).

After propensity score matching, 1750 patients remained in each group. The details of patients’ characteristics are depicted in Table 1. The risk of in-hospital mortality was significantly increased (OR 0.65, 95%CI 0.43–0.97, *p* = 0.03), and remained after the multivariable logistic regression (OR 0.58, 95%CI 0.35–0.98, *p* = 0.04) (Fig. 2).

## Discussion

This data based on a large real-world prospective registry, showed that TA decreased the risk of mortality among patients with STEMI that underwent P-PCI after matching the patients’ characteristics for the likelihood of being treated with TA. In Portugal we observed an increase in the use of TA but more than 50% of P-PCI evaluated were performed without this technique. It is possible that in some case TA was used as a bail-out procedure because the use of inhibitors of GpIIbIIIa was higher in the TA group. Our results are distinct from those obtained in largest RCTs, the TASTE and TOTAL trials [11, 12]. Despite the matching and the multivariable adjustments, our data is still retrospective, underpowered and with possible residual cofounding bias.

Improving the microvascular perfusion in patients with STEMI is attractive, particularly when large macroscopic thrombi are retrieved. It provides the feeling that operators interfere directly with the pathophysiologic mechanics and improve the prognostic of patients. The RCT data show that routine thrombus aspiration does not seem to influence the mortality of patients with STEMI. However, our data suggests that ‘selective’ TA may be useful to improve outcomes in patients with STEMI. The recognition of the type of patients in which TA is likely to be successful may be the key to the obtained results.

Due to limited data accuracy for other outcomes, our study only covered the in-hospital mortality. Similarly to the results regarding the short-term outcomes, the major trials also showed that TA did not improve 1-year outcomes [13, 14]. The results of observational data are heterogeneous. In a cohort with more than 10,000 patients with STEMI (about 3500 of STEMI patients treated with TA and P-PCI), TA did not show improvement in the risk of mortality [15]. Inversely, other observational studies showed that a ‘selective’ TA in P-PCI can improve outcomes, mortality included [16, 17]. The best available evidence, based on RCTs, is robust for not using ‘routinely’ the TA in P-PCI but do not preclude the use of TA in selected cases, as occurs with other interventions available for acute cardiac care setting [18].

In this cohort the rates of stroke were very low, and no differences were found between the groups, despite the cumulative evidence regarding this adverse event which is known to have a small increase in the absolute risk with TA [19].

### Limitations

Our results are limited because this is a consecutive all-comers procedural registry that has inherently limitations due to the lack of randomization and blinding effect. Despite the adjustment to multiple clinical and angiographic characteristics, it is worth noting that the data may not adjust to all potential confounders and thus increase the risk of bias in the analyses. The propensity score matching partially improves some of these limitations. However, this matching occurs with a decrease of the sample size which increases the risk of type II errors [20, 21]. Unfortunately, there are no data regarding the types of devices used in the TA as well as additional details of operators and procedures, which also hampers the conclusions.

## Conclusion

The use of thrombus aspiration did not have a significant impact in the short-term prognosis of STEMI patients that underwent primary percutaneous coronary intervention in the whole cohort. The results of the propensity-score matched cohort suggests a potential role for selective thrombus aspiration.

## Abbreviations

95%CI: 95% confidence interval
CNCDC: Centro nacional de colecção de dados em cardiologia
CNPD: Comissão nacional de proteccção de dados – national committee of data protection
GpIIbIIIa: Glycoproteins IIb/IIIa
OR: Odds ratio
P-PCI: Primary percutaneous coronary intervention
RCT: Randomized controlled trials
RNCI: Registo nacional de cardiologia de intervenção - portuguese registry on interventional cardiology
STEMI: ST-elevation myocardial infarction
TA: Thrombus aspiration
TASTE: Thrombus aspiration in myocardial infarction
TOTAL: A trial of routine aspiration thrombectomy with Percutaneous Coronary Intervention (PCI) versus PCI alone in patients with ST-Segment Elevation Myocardial Infarction (STEMI) Undergoing Primary PCI
